# Risk Factors for School Dropout in a Sample of Juvenile Offenders

**DOI:** 10.3389/fpsyg.2016.01993

**Published:** 2016-12-26

**Authors:** Asunción Fernández-Suárez, Juan Herrero, Beatriz Pérez, Joel Juarros-Basterretxea, Francisco J. Rodríguez-Díaz

**Affiliations:** ^1^Department of Psychology, University of OviedoOviedo, Spain; ^2^Núcleo en Ciencias Sociales y Humanidades, Universidad de la FronteraTemuco, Chile

**Keywords:** school dropout, juvenile delinquency, judicial records, risk factors, parental monitoring, irresponsibility, alcohol abuse, substances use

## Abstract

**Backgrounds:** The high rates of school dropout worldwide and their relevance highlight the need for a close study of its causes and consequences. Literature has suggested that school dropout might be explained by multiple causes at different levels (individual, family, school, and neighborhood). The aim of the current study is to examine the relation between individual (defiant attitude, irresponsibility, alcohol abuse, and illegal drugs use), family (educational figure absent and parental monitoring), school factors (truancy and school conflict) and school dropout.

**Method:** Judicial files of all juvenile offenders (218 males and 46 females) with a judicial penal measure in Asturias (Spain) in the year 2012 were examined. Multivariate logistic regression analyses were performed to estimate the relationships between school dropout and individual, family and school variables.

**Results:** As for the individual characteristics, results showed that school dropouts were more irresponsible than non-dropouts. Also they had higher rates of illegal drug use and alcohol abuse. Moreover, lack of parental monitoring emerged as a key predictive factor of school dropout, beyond the type of family structure in terms of the presence of both or only one educational figure. Finally, school factors did not show a significant relationship to school dropout.

**Conclusions**: These findings indicate that school dropout is a multidimensional process. School and family policies that emphasize the role of parental monitoring and prevent alcohol and substance abuse are recommended.

## Introduction

School dropout has been defined as leaving education without obtaining a minimal credential, most often a higher secondary education diploma (De Witte et al., 2013). Estimates of dropout rates seem to be higher in South and West Asia (43%) and sub-Saharian Africa (36%), while other geopolitical areas such as East Asia, and Europe show similar lower dropout rates (between 4 and 12%) (United Nations Educational, Scientific and Cultural Organization, 2012; European Commission Education Training, 2013). In Spain, where the present study is conducted, dropout rates are estimated as high as 22% (Andrei et al., 2012; Korhonen et al., 2014) with a greater incidence among males (26.6%). Although there is great diversity of standards by which school dropout and completion are measured across various studies (Cataldi et al., 2009), these figures illustrate the relevance of school dropout worldwide and ask for a close study of its causes and consequences.

Although it is often difficult to differentiate causes from consequences, youth who drop out from school are at increased risk for displaying socioemotional problems and engaging in delinquent and criminal behavior (Prevatt and Kelly, 2003; Lochner and Moretti, 2004; Bradshaw et al., 2008). Literature has also suggested that school dropout might be regarded as the last stage of a dynamic, cumulative and multidimensional process of school disengagement (Andrei et al., 2012; Bjerk, 2012; Fortin et al., 2013; Korhonen et al., 2014) in which multiple causes at different levels (individual, family, school, and neighborhood) might be explaining this phenomenon (Bronfenbrenner and Morris, 1998; Jimerson et al., 2000; Bradshaw et al., 2008; De Witte et al., 2013).

Among the individual risk factors, both internalizing and externalizing disorders have been claimed to have an influence on school dropout. Among the externalizing disorders, disruptive behavior seems to be the most impeding for educational attainment (Esch et al., 2014) whereas depression and anxiety are among the most studied internalizing problems (Tramontina et al., 2001; Kearney, 2008; Fortin et al., 2013; Quiroga et al., 2013). Patterson et al. (1989) suggested that children with early behavioral problems are at risk for developing academic problems and experiencing rejection from their prosocial peers, probably leading to connections with deviant peers and in turn engage in other maladjusted acts such as truancy, substance use, or possibly violent behavior (Bradshaw et al., 2008). Alternatively, students who conform to school rules tend to perform better in the classroom setting and are less likely to leave school early (Bradshaw et al., 2008). Moreover, disruptive behavior at school also influences parents' involvement and guidance (Dishion et al., 2004), as well as teachers' relationships with students (Hughes et al., 2001; Lewis et al., 2005; Settanni et al., 2015; Prino et al., 2016), thus exacerbating its effects on school performance (Tramontina et al., 2001; McGrath and Van Bergen, 2015).

Of special interest among the individual risk factors is substance abuse. The relationship between substance abuse and school dropout is among the most studied in official records (Esch et al., 2014), suggesting that students who are involved in drug or alcohol abuse are more likely to drop out from school (Battin-Pearson et al., 2000; Bradshaw et al., 2008; Patrick et al., 2016). For instance, Esch et al. (2014) found that students who continued their academic career had lower risk of becoming current drinkers than their peers who had dropped out from school. Likewise, those adolescents who began to use cannabis before the age of 16 were up to five times more likely to drop out of secondary school than their peers who did not consume any drugs (see also Harford et al., 2006; Crosnoe and Riegle-Crumb, 2007). However, possible mechanisms linking substance use with school dropout are unclear, ranging from cognitive and neurobiological deficits to learning difficulties and low academic performance (Townsend et al., 2007; DuPont et al., 2013; Goldberg-Looney et al., 2016; Park and Kim, 2016).

Among the family factors, socioeconomic status, family structure (De Witte et al., 2013), and the importance parents place on academic success (Bradshaw et al., 2008) have been related to school dropout. From a family socialization theoretical point of view, school performance and home environment are closely related (Battin-Pearson et al., 2000). For instance, stressful events such as parental divorce or family conflict might influence how a student behaves in and outside the classroom (Bradshaw et al., 2008). Beyond the existence of stressful events, family structure may also influence school dropout (De Witte et al., 2013). The empirical evidence show how children from single-parent households are more likely to dropout from school (Bridgeland et al., 2006; Román, 2013; Torres et al., 2015) and there is literature suggesting that family structure might influence socialization process (i.e., lack of rules) which in turn exacerbate its influence on school dropout. As Bridgeland et al. (2006) found, 38% of school dropouts believed that they did not have enough rules, making too easy to skip class or engage in activities outside of school. This lack of rules seemed to relate both to lack of order and discipline at school as to substance use and juvenile antisocial behavior (Cutrín et al., 2015). In this regard, Park and Kim (2016) found that living with parents has a protective effect against substance use, while low parental education level was associated with substance use, thus emphasizing the importance of family parental monitoring to reduce also the likelihood of substance use. Likewise, Guillén et al. (2015), in a sample of 1023 young students, found that parental monitoring would be able to strengthen resistance to peer pressure and therefore it can be expected to reduce alcohol consumption.

Regarding school factors, truancy has been identified in several studies as a risk factor for school dropout (Tramontina et al., 2001; Kearney, 2008; Ekstrand, 2015). According to Wilkins and Bost (2016), truancy might indicate that students are potentially disengaged from school and that a trajectory toward dropping out is likely. Truancy has been regarded as a resistance to the school culture (Zhang, 2007) which results in negative developmental outcomes such as deviant behaviors, crime and delinquency (Henry, 2007; Huck, 2011).

Of special interest for the current study is the fact that the literature has empirically linked school dropout and involvement with the justice system (De Witte et al., 2013). In this sense, literature has suggested that the reasons behind dropout are key to understand further engagement to delinquency: those who leave education early for personal reasons are probably more prone to display offending behavior than those leaving for economic reasons (Weerman, 2010).

The literature has traditionally analyzed dropout and delinquency in adult samples, mostly penitentiary samples, where crime has been studied as a result of school dropout and other school factors, such as school belonging (Lucero et al., 2015), learning-disabilities, attitudes toward school and scholastic experiences (Einat and Einat, 2015), school expulsion (Jaggers et al., 2016) or school mobility (Ou and Reinolds, 2010). For instance, Dianda (2008) found that 41% of inmates in state and federal prisons in the United States had less than a high school education, indicating that inmates who were dropouts were more likely to have served a prior sentence in prison and were more likely to have been sentenced when they were young. Similarly, Herrero et al. (2016), in a sample of 110 imprisoned males in Spain, found that most of them (60%) did not have secondary studies. Likewise, Einat and Einat (2015), in a sample of 89 adult inmates in Israel, found that those who dropped out of school early began their criminal activity at an earlier stage, suggesting that completing high school reduces the probability of incarceration (Lochner and Moretti, 2004).

To date, few studies have analyzed school dropout among juvenile offenders, despite its alarming rates of school dropout as compared to the juvenile general population (Andrei et al., 2012; Kim, 2012; Korhonen et al., 2014). Drawing from the reviewed literature, the current study examined the relation between individual (defiant attitude, irresponsibility, alcohol abuse and illegal drugs use), family (educational figure absent and parental monitoring), school factors (truancy and school conflict) and school dropout among juvenile offenders. The research question that motivated the present research was: do school dropouts and non-dropouts differ in their characteristics in the individual, family, and school contexts? Specifically, we analyze the presence of school dropout (defined as leaving school before or during their criminal career) among juvenile offenders taking into account individual, family, and school correlates that have been empirically found to be related to school dropout.

## Methods

### Participants

Participants of the study were 264 young offenders drawn from the population of convicted young offenders 14–18 years-old with a judicial penal measure in Asturias (Spain). The population consisted in 270 young offenders (218 males and 46 females). Six of them, however, did not have information about school dropout in their criminal records so they were not retained for further analyses. All participants had committed at least one criminal offense in the year 2012. Participants varied considerably in terms of the type of offense: 42.8% were generalist offenders—different type of offenses on various occasions—and 57.2% were specialist offenders—tendency to repeat the same offense over time—. Offenses committed most frequently were property offenses (73.9%), injuries (45.5%), offenses against public security (17%), offenses against public order (12.9%), threats (11.4%), and child to parent violence/bullying/dating violence (11.4%).

### Procedure

The researchers contacted the Juvenile Prosecutor of Asturias (Spain) and explained the objectives of the study. After access for the official records was granted, confidentiality of participants was guaranteed, according to the Organic Law 15/1999 on the Protection of Personal Data in Spain as well as the Declaration of Helsinki. The official records provided not only information about the criminal history of all participants but, also, their forensic evaluation. This evaluation was conducted by health professionals. The psychological, family, and school correlates were assessed through an in-depth evaluation of the multidisciplinary team of psychologists and counselors for each participant. The present paper is an empirical study, which was conducted with a quantitative methodology and a retrospective design.

### Measures

#### Outcome variable

Participants were divided into two groups: school dropouts (*n* = 128; 48.5%)—juvenile offenders who had left school before or during their criminal career—and non-dropouts (*n* = 136; 51.5%)—juvenile offenders who remain at school by the time they committed their last offense in 2012—. Response categories were 0 for non-dropout, and 1 for dropout.

#### Individual variables

*Psychological characteristics* of respondents were retrieved from official records. For this study, information about two individual characteristics was used: defiant attitude and irresponsibility. Defiant attitude measures whether the participant regularly rejected authority and showed trouble in compliance with rules, limits, schedules and orders or not (*n* = 120; 45.5% of them). Irresponsibility measures whether the participant was responsible for his/her behavior or not (*n* = 86; 32.6% of them was described by professionals as irresponsible). *Substance use and abuse*. Substance use and abuse (including cannabis, cocaine, heroin, inhalants, amphetamines, etc.) was assessed as present if participant reported having use substances 4 or more times a week. While 15.9% (*n* = 42; 12 missing cases) of juvenile delinquents abuse alcohol, 61.4% (*n* = 162; 12 missing cases) of them use illegal drugs.

#### Family variables

Family structure and parental monitoring were family variables of the study. *Family structure* was measured as the presence of both parents in child-rearing or not. In 183 cases (69.3%) the father/mother had been absent. *Parental monitoring* was measured as the presence of clear limits and rules about the behavior of participants at home. In 112 cases (42.4%) there were not clear rules.

#### School variables

Truancy and conflict at school were the school variables of the study. *Truancy* was measured as the tendency observed for each participant of missing school. Truancy was considered to be present if the student was absent from class without informed consent for 3 or more days within a 4-week period, or for 10 or more days within a 6-month period. In 146 participants (55.3%) it was found a tendency to miss school regularly. *School conflict* measured whether there was a history of conflict with teachers, peers or school equipment or not. In 110 participants (41.7%) it was observed a history of conflict.

### Statistical procedures

Multivariate logistic regressions were conducted to determine the relationship between school dropout and individual, family and school variables. Chi-squared tests were first conducted for each set of variables (individual, family, and school) to analyze their bivariate associations with dropouts, and Cramer's V was used as a measure of effect size for this association.

## Results

Multivariate logistic regression analyses tested whether dropouts showed statistically significant differences compared to non-dropouts in the variables of the study. To do so, individual, family, and school variables were entered into the equation in a sequential fashion to further analyze the joint contribution of each variable of the study. Model 1 incorporated individual psychological variables. Model 2 jointly analyzed all the individual variables, including alcohol and drugs abuse to Model 1. Model 3 incorporated family variables to the previous Model 2. Final Model 4, included school variables to Model 3. For model fit evaluation, Nagelkerke R^2^ was estimated for each model. Odds ratios [Exp. (b)] and their 95% confidence intervals were used to test for statistical significance of each variable of the study on the outcome variable. Results for all models are presented in Table 1. Also, sample size, percentage, Chi-squared and Cramer's V tests for each set of variables (individual, family, and school) in each group of juvenile delinquents are presented in Tables 2–4.

**Table 1:** **Multivariate logistic regression analysis for individual (psychological and alcohol/drugs abuse), family and school variables predicting school dropout**.

	**Model 1 Nagelkerke *R*^2^ = 0.098**		**Model 2 Nagelkerke *R*^2^ = 0.182**		**Model 3 Nagelkerke *R*^2^ = 0.220**		**Model 4 Nagelkerke *R*^2^ = 0.223**	
	**Exp. (b)**	**IC 95%**	**Exp. (b)**	**IC 95%**	**Exp. (b)**	**IC 95%**	**Exp. (b)**	**IC 95%**
Irresponsibility	2.116^**^	1.204–3.718	1.972^*^	1.100–3.535	2.019^*^	1.101–3.701	1.981^*^	1.075–3.652
Defiant attitude	2.082^**^	1.229–3.525	1.488	0.846–2.616	1.276	0.711–2.293	1.199	0.652–2.207
Alcohol abuse			2.371^*^	1.108–5.072	2.142^+^	0.986–4.654	2.180^*^	1.003–4.739
Illegal drugs use			2.639^***^	1.467–4.745	2.442^***^	1.346–4.431	2.373^**^	1.288–4.370
Educational figure absent					1.663^+^	0.910–3.039	1.585	0.858–2.930
Parental monitoring					0.505^*^	0.285–0.895	0.522^*^	0.290–0.940
Truancy							1.148	0.610–2.162
School conflict							1.191	0.663–2.140

*** p < 0.001;

** p < 0.01;

* p < 0.01;

+ *p < 0.10*.

**Table 2:** **Sample size, percentage, χ^2^ and Cramer's V test on individual variables and drugs use**.

**Individual variables**	**Dropouts (*n* = 128)**	**Non-dropouts (*n* = 136)**	**χ^2^**	**Cramer's V**
Defiant attitude			12.540^***^	0.23^***^
Yes (n)	73	47		
Yes (%)	57	34.6		
Irresponsibility			11.318^***^	0.21^***^
Yes (n)	55	31		
Yes (%)	43	22.8		
Alcohol abuse^∧^			9.971^***^	0.21^***^
Yes (n)	30	12		
Yes (%)	24.8	9.2		
Illegal drugs use^∧^			19.345^***^	0.28^***^
Yes (n)	95	67		
Yes (%)	78.5	51.1		

∧ 12 missing cases;

*** *p < 0.001*.

**Table 3:** **Sample size, percentage, χ^2^ and Cramer's V test on family variables**.

**Family variables**	**Dropouts (*n* = 128)**	**Non-dropouts (*n* = 136)**	**χ^2^**	**Cramer's V**
Educational figure absent			3.271^+^	0.12^+^
Yes (n)	96	87		
Yes (%)	75	64		
Parental monitoring			15.505^***^	0.25^***^
Yes (n)	38	74		
Yes (%)	29.7	54.4		

*** p < 0.001;

+ *p < 0.10*.

**Table 4:** **Sample size, percentage, χ^2^ and Cramer's V test on School variables**.

**School factors**	**Dropouts (*n* = 128)**	**Non-dropouts (*n* = 136)**	**χ^2^**	**Cramer's V**
Truancy			7.040^**^	0.17^***^
Yes (n)	82	64		
Yes (%)	64.1	47.1		
School conflict			3.205^+^	0.12^+^
Yes (n)	61	49		
Yes (%)	47.7	36		

*** p < 0.001;

** p < 0.01;

+ *p < 0.10*.

Results for Model 1, which incorporated only individual psychological variables, showed that both being irresponsible and defiant increased the odds ratios of having dropped out from school. The inclusion of substance use and abuse variables in Model 2, however, removed the statistical significance of defiant attitude and school dropout, in spite of Chi squared test showed that dropouts display significantly a more defiant attitude than non-dropouts (see Table 2). In this Model 2, having being described as irresponsible by professionals and reporting heavy alcohol consumption and illegal drugs use were positively related to having dropped out from school. The effect of defiant attitude on school dropout seemed to be completely explained by the presence of alcohol abuse and illegal substance consumption. As for results of Model 3, which incorporated family variables, the existence of parental monitoring in the family was negatively related to school dropout, suggesting that those participants with clear limits and rules at home presented lower odds ratio of dropping out from school, regardless their individual characteristics and patterns of substance use and abuse. The absence of a family educational figure did not seem to have an effect beyond the existence of parental monitoring in the family. Finally, Model 4 showed that school variables did not influence school dropout beyond individual and family variables. Although both truancy and school conflict showed a bivariate positive relationship with school dropout (see Table 4), this relationship was non-significant after taking into account the individual and family variables of the study.

Overall, results from final Model 4 suggested that individual characteristics such as being irresponsible, substance use and alcohol abuse, and lack of parental monitoring in the family were key to understand the existence of school dropout among participants. Otherwise stated, juvenile delinquents of the study who stayed at school during the compulsory years of education were assessed by professionals as more responsible, low on substances consumption and alcohol abuse, and more supervised in their family.

## Discussion

In the present study school dropout was examined from a multidimensional approach, where individual, family and school (Andrei et al., 2012; Bjerk, 2012; Fortin et al., 2013; Korhonen et al., 2014) correlates of school dropout were analyzed among juvenile offenders, a population with a high risk of school dropout (Lochner and Moretti, 2004; Dianda, 2008; Ou and Reinolds, 2010; Andrei et al., 2012; De Witte et al., 2013; Korhonen et al., 2014; Einat and Einat, 2015; Lucero et al., 2015; Herrero et al., 2016; Jaggers et al., 2016). The official records of 264 juvenile delinquents were used to analyze the individual, family, and school correlates of school dropout.

As for the differences in their individual characteristics, the school dropouts seemed to be more irresponsible than non-dropouts. Students who did not comply with rules, limits, schedules and orders (i.e., they arrive late at school or return late from playtime) were at risk for school dropout. This finding would support the idea that a disruptive behavior is the most impeding for educational attainment (Patterson et al., 1989; Bradshaw et al., 2008; Esch et al., 2014). Although school dropouts and non-dropouts differ in their defiant attitudes, the effect of this psychological characteristic on school dropout seemed to be completely explained by the presence of alcohol abuse and illegal drugs consumption. This result support the idea that substances use is associated with deviant behaviors (Townsend et al., 2007) and externalizing symptoms (Meier et al., 2015).

Also, alcohol abuse and substance use were predictive of higher rates of school dropout. This finding would be consistent with research showing that both alcohol and substance dependence may increase the likelihood of school dropout (Battin-Pearson et al., 2000; Harford et al., 2006; Crosnoe and Riegle-Crumb, 2007; Townsend et al., 2007; Bradshaw et al., 2008; Esch et al., 2014; Patrick et al., 2016). Alcohol abuse and substance use have direct consequences on individual characteristics that relate to deviant behaviors, externalizing symptoms, psychological problems and risky behaviors (Townsend et al., 2007; Meier et al., 2015; Park and Kim, 2016), on cognitive process leading poor planning, impaired executive functioning or attention deficits (DuPont et al., 2013), and even on academic motivation (Park and Kim, 2016), being their effects incompatibles with keeping in school. Likewise, adolescent alcohol and drug users often reduce the number of hours committed to studying, completing homework assignments, and attending school, engaging in a vicious cycle which cause loss of interest in pursuing academic goals (DuPont et al., 2013).

Regarding family variables, lack of parental monitoring emerged as a key predictive factor of school dropout, beyond the type of family structure (absence of educational figures). These results suggest that, indeed, there would be family socialization differences in each group: parents of school dropouts seem to not clearly put limits and rules (i.e., they do not control the arrival time from school, or do not know about recreational activities of adolescents). This finding would be consistent both with family socialization theory (Battin-Pearson et al., 2000) and with the empirical evidence linking lack of rules and school dropout (Bridgeland et al., 2006; Bradshaw et al., 2008; De Witte et al., 2013; Román, 2013; Torres et al., 2015). The existence of family parental monitoring, however, seems to be more relevant than the absence of parents in child rearing, according to our data. Thus, parental monitoring seemed to be associated with a reduction of school dropout rates, whether both parents of these participants were present or not.

Once individual and family variables were taken into account, school-related variables such as truancy and the presence of conflicts with teachers and peers at school did not show a significant relationship with school dropout. This result contradicts research showing truancy as a risk factor for school dropout (Tramontina et al., 2001; Kearney, 2008; Ekstrand, 2015); however, it could be explained because most of these studies do not take into account other potentially relevant influences in the psychological and family realms, as our study shows.

### Implications for practice

Results from our study clearly highlight the role that the individual and family characteristics play on the explanation of school dropout thus pointing out where prevention and intervention efforts should put the accent on. In this sense, it seems that school dropouts of our study would benefit from both school and family policies that emphasize the role of supervision of adolescents. For instance, dealing with the irresponsible nature of participants would probably reduce school dropout rates (i.e., a closer control of time schedules, monitoring the homework or their recreational activities). Likewise, a greater prevention and intervention effort aimed to provide parents with educational and communicational tools that allow them to better monitor adolescents would probably lead to a reduction in school dropout rates. In addition, parents and teachers might play a key role on prevention of substance abuse, in so far as they promote alternative recreational activities which are incompatibles with consumption (such as sport) and develop tools that help them to early detection of substance abuse. For instance, prevention efforts directed to address substance use and related problems among students who are experiencing academic difficulties would be needed. Also, continued care monitoring systems to track their progress and to provide more intensive supports are warranted while strategies such as punitive methods (i.e., student expulsion) should be avoided (DuPont et al., 2013). Rather, parents should monitor and supervise adolescent activities, expressing disapproval of drinking and other drug use and communicating a zero-tolerance message (Prevatt and Kelly, 2003; Dick and Hancock, 2015).

### Strengths and limitations

The study presents strengths and potential limitations. Among the strengths, participants of the study were representative of the population of juvenile offenders of Asturias (Spain), which might add generalizability of the study findings. As for the potential limitations, given the cross-sectional nature of the data used other alternative explanations of the observed relationships in our study are also possible. Thus, although we claimed that individual, family, and school variables were predictive of school dropout, the reverse might also be true: school dropout influenced individual, family and school variables. In this sense, the variables used in our study might be seen both as causes and consequences of school dropout thus warranting new research that takes into account the temporal dimension (i.e., follow-up studies). Also, participants of the study were mainly male (about 80%), so generalization of results across sex might not be tenable. Although participants of the study were almost the population of convicted young offenders 14–18 years-old with a judicial penal measure in Asturias (98% of those convicted), future research would benefit from a greater representation of female participants to analyze different potential paths for female school dropout.

Results of the present study, however, are in line with previous research about the role that individual, family and school variables have on school dropout, so we are confident that our findings might help to a better understanding of school dropout among juvenile offenders.

## Author contributions

All authors jointly co-authored the content.

### Conflict of interest statement

The authors declare that the research was conducted in the absence of any commercial or financial relationships that could be construed as a potential conflict of interest.
